# Nymphaeol-A Isolated from Okinawan Propolis Suppresses Angiogenesis and Induces Caspase-Dependent Apoptosis via Inactivation of Survival Signals

**DOI:** 10.1155/2013/826245

**Published:** 2013-04-24

**Authors:** Ikumi Tsuchiya, Takahiro Hosoya, Motoko Ushida, Kazuhiro Kunimasa, Toshiro Ohta, Shigenori Kumazawa

**Affiliations:** ^1^Department of Food and Nutritional Sciences, University of Shizuoka, 52-1 Yada, Suruga-ku, Shizuoka 422-8526, Japan; ^2^Cancer Chemotherapy Center, Japanese Foundation for Cancer Research, 3-10-6 Ariake, Koto-ku, Tokyo 135-8550, Japan

## Abstract

Propolis, a resinous substance that honeybees collect to protect their beehive from enemies, is reported to have various biological activities. In our screening program to search for antiangiogenic compounds from propolis, the ethanol extracts of Okinawan propolis (EEOP) showed significant antiangiogenic activities in a tube formation assay with human umbilical vein endothelial cells (HUVECs) *in vitro* at 3.13 **μ**g/mL and chorioallantoic membrane (CAM) assay *in vivo* at 25 **μ**g/egg. To elucidate the active compounds of EEOP and their mode of action, we isolated some prenylated flavonoids from EEOP and found that nymphaeol-A had the strongest antiangiogenic activity among them. Nymphaeol-A significantly reduced *in vivo* neovessel formation in the CAM assay at 25 **μ**g/egg. At the molecular level, nymphaeol-A markedly inactivated mitogen-activated protein kinase/ERK kinase 1/2 (MEK1/2) and extracellular signal-regulated kinase 1/2 (ERK1/2), whose molecular activations signal new vessel formation in HUVECs. In addition, nymphaeol-A dose- and time-dependently induced caspase-dependent apoptosis in tube-forming HUVECs. Taken together, nymphaeol-A was shown to inhibit angiogenesis at least in part via inactivation of MEK1/2–ERK1/2 signaling and induction of caspase-dependent apoptosis. Okinawan propolis and its major component, nymphaeol-A, may be useful agents for preventing tumor-induced angiogenesis.

## 1. Introduction

Angiogenesis is defined as the process of forming new blood vessels from preexisting ones. Folkman first observed that the angiogenesis is required for tumor growth in 1971 [1]. Tumor-induced neovessels carry oxygen and nutrients into tumor tissues and function as the primary path of metastasis. Cutting off the blood supply of oxygen and nutrients to solid tumors represents a useful antiangiogenic therapy for tumors. Bevacizumab, a monoclonal antibody that binds to vascular endothelial growth factor (VEGF), was developed as an antiangiogenic drug for colon cancer treatment. In recent years, various antiangiogenic inhibitors have been developed, used in clinical trials, and approved for a number of cancer treatments. Antiangiogenic treatment may be useful in the treatment and prevention of cancer progression.

Propolis, a resinous substance collected by honeybees from the buds and exudates of certain trees and plants, is stored inside their hives and has been used in folk medicines from ancient times in many regions. It has been reported to have various biological activities such as antioxidant [2], antibacterial [3], antiviral [4], antifungal [5], anti-inflammatory [6], and anticancer [7] activities. In general, propolis is known to have various chemical compositions depending on the vegetation at the site of its collection. For example, propolis from Europe and China contains many kinds of flavonoids and phenolic acid esters. In contrast, propolis from Brazil contains terpenoids and prenylated derivatives of *p*-coumaric acids [8–11]. In our previous studies, we reported that artepillin C, a major phenolic component of Brazilian propolis, suppressed tumor-induced angiogenesis [12].

**Figure 1:**
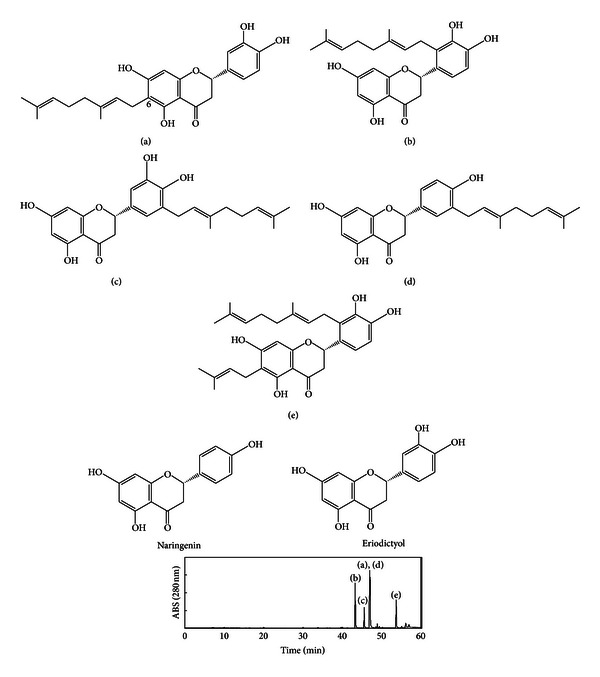
Structures of the main components isolated from Okinawan propolis and HPLC profile of EEOP. (a) Nymphaeol-A; (b) nymphaeol-B; (c) isonymphaeol-B; (d) 3′-geranyl-naringenin; (e) nymphaeol-C; naringenin, and eriodictyol. HPLC conditions: column, Capcell Pak UG120 C18 column (250 × 4.6 mm i.d.; Shiseido, Tokyo, Japan); flow rate, 1.0 mL/min; detection, 280 nm; solvent, (A) water with 0.1% trifluoroacetic acid (B) acetonitrile with 0.1% trifluoroacetic acid (B); gradient, 10–100% (B) in 60 min.

Through our programs of revealing the constituents of the worldwide propolis, we have analyzed the components of a propolis collected in Okinawa, the southernmost prefecture of Japan [13–16]. It mainly contains prenylated flavonoids, which are called nymphaeols, analogs of naringenin, and/or eriodictyol (Figure 1), and the ethanol extracts of Okinawan propolis (EEOP) and the isolated prenylated flavonoids show strong antioxidant and antibacterial activities [13, 14]. In addition, they show cytotoxicity against some cancer cell lines as well as antimalarial activity [17]. Nymphaeols, the main prenylated flavonoids isolated from Okinawan propolis, have been reported to originate from *Macaranga tanarius* [18, 19]. We have already revealed that the plant origin of Okinawan propolis is *M. tanarius* and that the major components of Okinawan propolis are nymphaeols-A (a) and -B (b) (Figure 1) [14].

In the present study, our aims were to investigate the *in vivo* antiangiogenic effects of EEOP and its two main prenylated flavonoids, nymphaeols-A and -B, by chorioallantoic membrane (CAM) assay using fertilized chicken eggs. Furthermore, we evaluated their modes of action of antiangiogenic effects by investigating the inhibition of tube formation, as well as changes in survival signals and apoptotic pathways using human umbilical vein endothelial cells (HUVECs).

## 2. Materials and Methods

### 2.1. Materials and Chemicals

Medium 199 was purchased from Sigma (St. Louis, MO, USA). Medium MCDB-104 was purchased from Nihon Pharmaceutical (Tokyo, Japan). Fetal bovine serum (FBS) was purchased from Moregate (Brisbane, Australia). Cellgen was obtained from Koken (Tokyo, Japan). Epidermal growth factor (EGF) was purchased from BD Biosciences (Bedford, MA, USA). Human basic Fibroblast Growth Factor (recombinant) was purchased from Wako Pure Chemicals Industries (Osaka, Japan).

Propolis used for this study was collected as a crude material by beekeepers of Aragaki bee farm, Naha, Okinawa, Japan, in June 2010. A voucher sample of the propolis (HSRCTU-BPC PR0002/2010) has been deposited at Bee Products Collection, Honeybee Science Research Center, Tamagawa University, Tokyo, Japan. Okinawan propolis collected from Okinawa, Japan, was extracted with ethanol by stirring overnight at room temperature. After extraction, the solvent was evaporated *in vacuo* to give EEOP. The nymphaeols used in the experiments were purified from the ethanol extracts of Okinawan propolis by column chromatography including HPLC as described previously [16]. The purity of each compound was >98% using ^1^H NMR spectrum.

### 2.2. Cell Culture

HUVECs were grown in HUVEC growth medium (MCDB-104 medium supplemented with 10 ng/mL EGF, 100 *μ*g/mL heparin, 100 ng/mL endothelial cell growth factor, and 10% FBS) as previously reported [20] and incubated at 37°C under a humidified 95/5% (v/v) mixture of air and CO_2_. The cells were seeded on plates coated with 0.1% gelatin and allowed to grow to subconfluence before experimental treatments.

### 2.3. CAM Assay

CAM assay was performed as described previously [21]. Briefly, fertilized chicken eggs were incubated at 37°C. On incubation day 3, a small window was opened in the shell and 4 mL of albumen was removed. After incubation at 37°C, the 5-day-old CAM was treated with various doses of samples and incubated at 37°C for another 2 days. An appropriate volume of white emulsion was injected into CAM to clearly visualize the vascular network. Observation of the vascular networks was carried out to evaluate antiangiogenic activity. Retinoic acid (5 nmol/egg) was used as a positive control.

### 2.4. Tube Formation Assay

Capillary tube-like structures formed by HUVECs in collagen gel were prepared as previously described with slight modifications [20]. Collagen gels were made by Cellgen (type I collagen). 200 *μ*L of collagen solution (0.21% in Medium-199) was poured into wells of a 24-well plate, and the plates were incubated at 37°C for 30 min to solidify gels. HUVECs (6.0 × 10^4^ cells/cm^2^) in MCDB-104 medium with 0.5% FBS were seeded onto collagen-coated wells and left at 37°C in a 5% CO_2_ incubator for 1 h to attach to the collagen gel. After removing the medium, 150 *μ*L of the collagen solution was overlaid and subjected to gelation as described above. Subsequently, 650 *μ*L of MCDB-104 with 0.5% FBS supplemented with 10 ng/mL bFGF, 8 nM/mL phorbol 12-myristate 13-acetate (PMA), together with various concentrations of samples, was added to the wells and incubated for up to 36 h. The resulting web-like capillary structure was viewed under a microscope, and images were captured using an Olympus C-4040ZOOM digital camera (Olympus, Tokyo, Japan).

### 2.5. Western Blot Analysis

After experimental treatment with various doses of samples, cells embedded 3D in collagen gel were treated with SDS sample buffer (50 mM Tris-HCl (pH 6.8), 2% SDS, 5.88% 2-mercaptoethanol, 10% glycerin, 1 mM *β*-glycerophosphate, 2.5 mM sodium pyrophosphate, 1 × phosphatase inhibitor cocktail 1, 1 × phosphatase inhibitor cocktail 2, and 1 × protease inhibitor cocktail 1) and boiled for 10 min. Each sample was electrophoresed in 6–12% SDS-polyacrylamide gels and then transferred to a Hybond-ECL nitrocellulose membrane (GE Healthcare, Buckinghamshire, UK). Immunoreactive protein bands were visualized using the ECL plus detection system with an ECL Minicamera (GE Healthcare). Results were obtained from three independent experiments.

### 2.6. Caspase Inhibition by z-VAD-fmk

HUVECs in MCDB-104 with 0.5% FBS supplemented with 10 ng/mL bFGF, 8 nM/mL PMA on 96-well plates were treated with both 1.56–6.25 *μ*g/mL nymphaeol-A and 10 *μ*M z-VAD-fmk (Promega, WI, USA), an irreversible pan-caspase inhibitor, for 12 h. Caspase-3/7 activity was examined by a Caspase-Glo Assay (Promega) according to the manufacturer's protocol. Luminescence was measured using a Flex Station II system (Molecular Devices, Inc., CA, USA).

### 2.7. ERK1/2 Activation by Angiotensin II

HUVECs in MCDB-104 with 10% FBS on 96-well plates were pretreated with 1 *μ*M angiotensin II (Sigma) for 2 h. Pretreated HUVECs were incubated with 3.13 *μ*g/mL nymphaeol-A. Caspase-3/7 activity was examined by a Caspase-Glo Assay (Promega) according to the manufacturer's protocol. Luminescence was measured using a Flex Station II system (Molecular Devices, Inc.).

### 2.8. Statistical Analysis

All data was expressed as means ± SEM of at least three independent experiments. Comparisons between control and treatments were performed using Student's unpaired *t-*test (*P < 0.05).

## 3. Results

### 3.1. Antiangiogenic Activity of EEOP *In Vitro* and *In Vivo*

We first examined the effects of ethanol extracts of Okinawan propolis (EEOP) in tube formation assay as an *in vitro* model and CAM assay as an *in vivo* model.

The tube formation assay was carried out using HUVECs and the extent of capillary network formation was evaluated. Regarding the CAM assay, the fertilized chicken egg model is a well-known animal model in biological and pharmaceutical research in terms of simplicity and low cost. Because CAMs have a very dense capillary network, CAM assays are commonly used to study *in vivo* angiogenesis and its inhibition in response to different compounds. In the CAM assay, the antiangiogenic activities of samples were judged on day 7.

**Figure 2:**
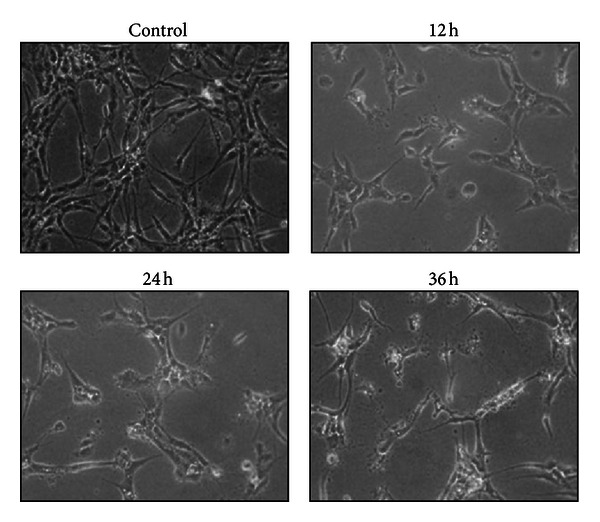
EEOP inhibits tube formation in HUVECs. HUVECs were sandwiched between two layers of collagen gel and induced to form blood-vessel-like tubes. HUVECs were treated with 3.13 *μ*g/mL EEOP for the indicated time periods.

**Figure 3:**
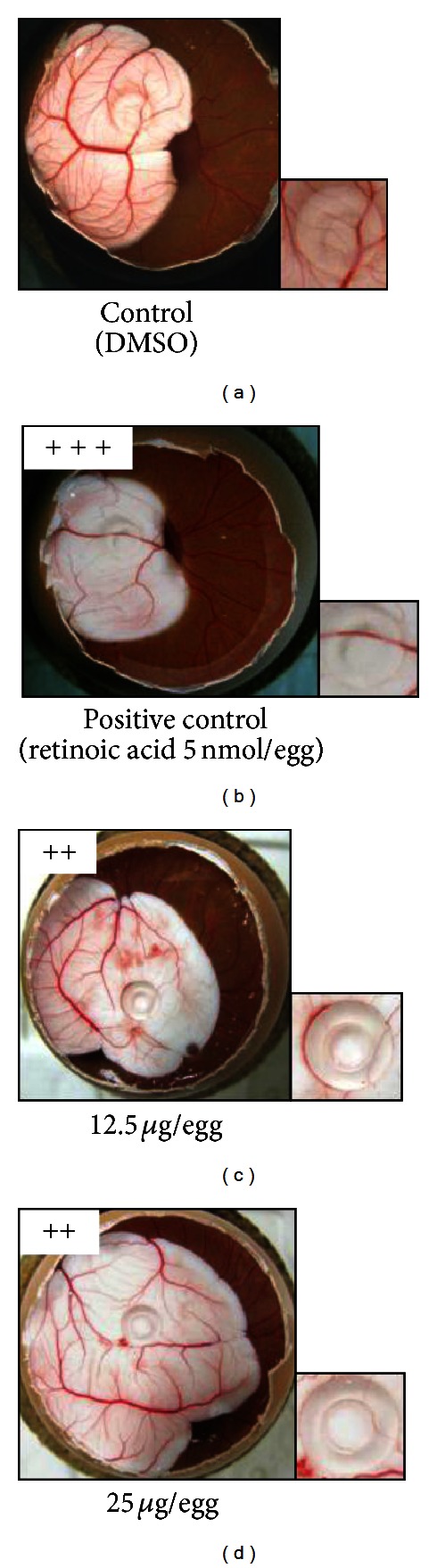
EEOP inhibits angiogenesis *in vivo. *EEOP showed antiangiogenic effects in CAM assay. (a) Control; (b) 5 nmol/egg Retinoic acid; (c) 12.5 *μ*g/egg EEOP; (d) 25 *μ*g/egg EEOP.

As shown in Figures 2 and 3, EEOP inhibited angiogenesis in both *in vitro* and *in vivo* models. EEOP inhibited the formation of capillary networks by HUVECs at 3.13 *μ*g/mL, time-dependently, and also suppressed embryonic angiogenesis at 12.5 and 25 *μ*g/egg.

### 3.2. Antiangiogenic Activity *In Vitro* of Nymphaeols and Their Derivatives Isolated from EEOP

EEOP showed antiangiogenic activity in tube formation and CAM assays. To confirm the active compounds, five prenylated flavonoids were isolated and purified by repeated column chromatographies. Figure 1 shows the chemical structures isolated from EEOP and the HPLC chromatogram of EEOP.

**Figure 4:**
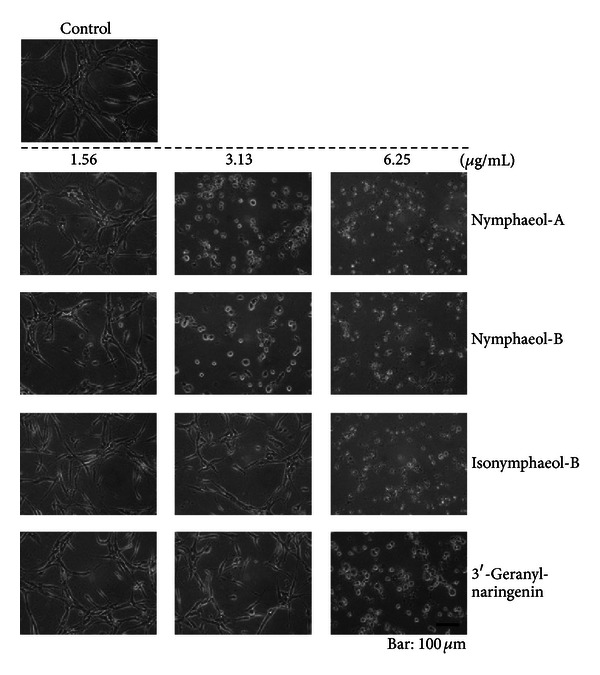
Nymphaeol derivatives inhibit angiogenesis *in vitro*. HUVECs were sandwiched between two layers of collagen gel and induced to form blood-vessel-like tubes. HUVECs were treated with the indicated concentrations of each compound for 36 h.

These compounds were tested for antiangiogenic effects in tube formation assay (Figure 4). Among them, nymphaeol-A, the main component of EEOP, showed the strongest activity compared with that of other prenylated flavonoids. We also evaluated the anti-tube-forming activity of naringenin and eriodictyol, which possess the same main skeleton as these prenylated derivatives, but neither showed any detectable activity at 25 *μ*g/mL (data not shown). These results indicate that it is important to have a geranyl moiety at the C-6 position for antitube-formation activity.

### 3.3. Antiangiogenic Activity *In Vivo* of Nymphaeols-A and -B

**Figure 5:**
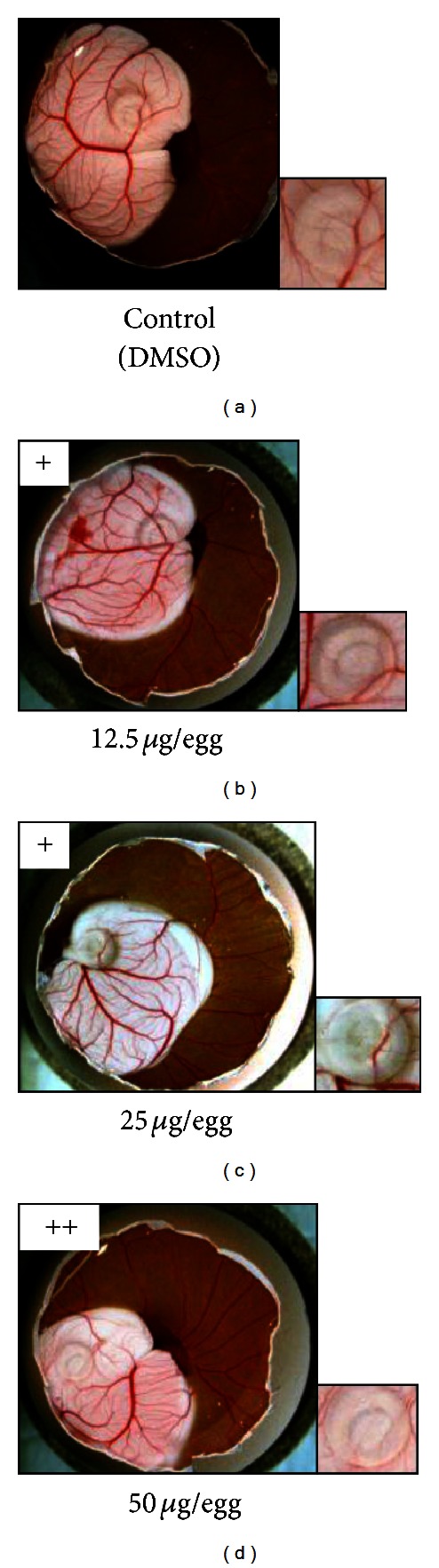
Nymphaeol-A inhibits angiogenesis *in vivo*. Nymphaeol-A showed strong antiangiogenic effects in CAM assay. (a) Control; (b) 12.5 *μ*g/egg nymphaeol-A; (c) 25 *μ*g/egg nymphaeol-A; (d) 50 *μ*g/egg nymphaeol-A.

**Figure 6:**
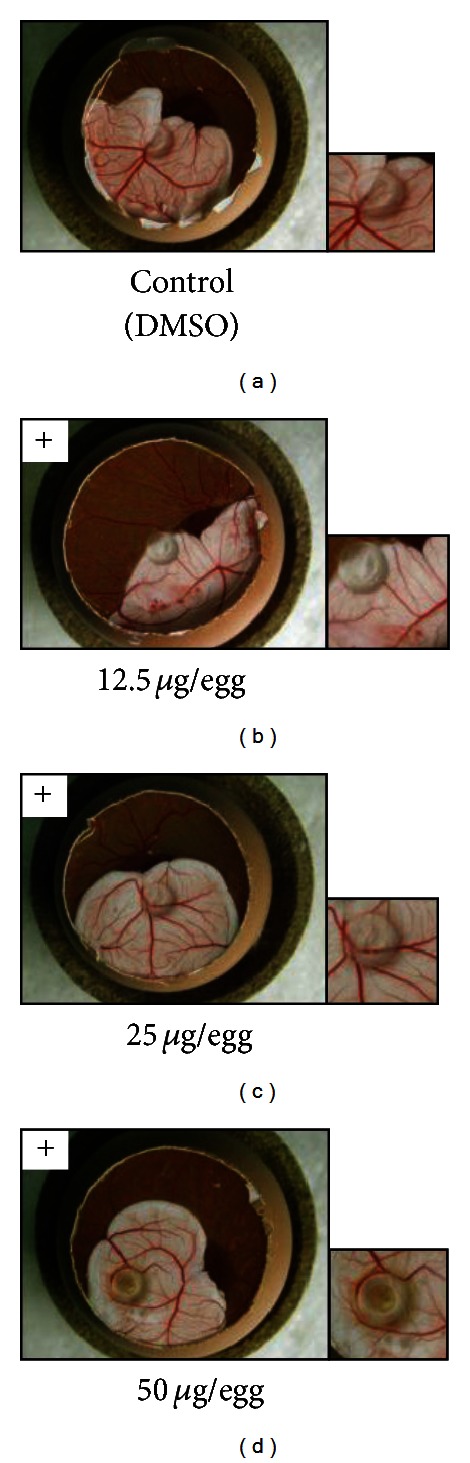
Nymphaeol-B inhibits angiogenesis *in vivo*. Nymphaeol-B showed an antiangiogenic effect in CAM assay. (a) Control; (b) 12.5 *μ*g/egg nymphaeol-B; (c) 25 *μ*g/egg nymphaeol-B; (d) 50 *μ*g/egg nymphaeol-B.

We decided to evaluate *in vivo* antiangiogenic activities of nymphaeols-A and/or -B because they were the main components of EEOP and could be the cause of the antiangiogenic activity of EEOP using the CAM assay. As shown in Figure 5, nymphaeol-A inhibited embryonic angiogenesis at 12.5 *μ*g/egg. Nymphaeol-B also showed antiangiogenic activity, but the activity of nymphaeol-B was weaker than that of nymphaeol-A (Figure 6). This *in vivo* result was consistent with the findings in the *in vitro* tube-forming model. Thus, the importance of having a geranyl moiety at the C-6 position for antiangiogenic activity has been shown both *in vivo* and *in vitro.*

### 3.4. Caspase Activation and Apoptosis Induction by Nymphaeol-A

To examine whether apoptosis was involved in antiangiogenic effects of nymphaeol-A, tube-forming HUVECs were treated with nymphaeol-A at various concentrations. After treatment for different time periods, activation of caspase-3, one of the effector caspases of apoptosis, was evaluated by western blotting analysis.

**Figure 7:**
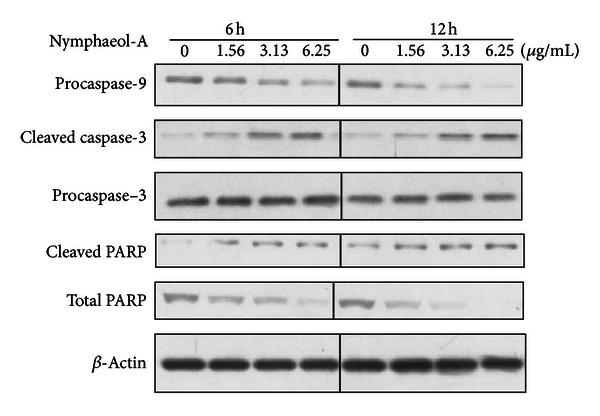
Nymphaeol-A activates caspases in tube-forming HUVECs. HUVECs were treated with 0.4% DMSO (0 *μ*g/mL) or nymphaeol-A at the indicated concentrations for 6 h and 12 h, respectively. Changes in the levels of caspase-9, caspase-3, and PARP were analyzed by western blotting. Each experiment was repeated at least three times and representative data are shown.

As shown in Figure 7, treatment with nymphaeol-A significantly increased the level of cleaved caspase-3, the active form, in a time- and a concentration-dependent manner, and decreased the level of procaspase-3. To identify the signal cascade that leads to caspase-3 activation, we analyzed the activation of caspase-9 by western blotting. The signal for procaspase-9 was decreased after treatment with nymphaeol-A in a time- and concentration-dependent manner (Figure 7). PARP is a 116-kDa nuclear poly (ADP-ribose) polymerase known to be a molecular marker of apoptosis. The band for cleaved PARP was detected after treating cells with nymphaeol-A, also in a time- and concentration-dependent manner (Figure 7). These results confirmed that nymphaeol-A induces apoptosis in tube-forming HUVECs.

**Figure 8:**
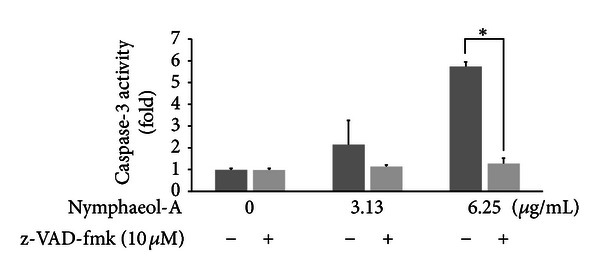
z-VAD-fmk reverses apoptosis induction by nymphaeol-A. HUVECs were treated with nymphaeol-A at the indicated concentrations and 10 *μ*M z-VAD-fmk, a pan-caspase inhibitor, for 12 h. Caspase-3/7 activity was measured using the Caspase-Glo Assay Kit by luminescence assays as described in the text. *P < 0.05, as compared with the control group.

We focused on the caspase-dependent apoptosis induced by nymphaeol-A. We examined the contribution of nymphaeol-A to caspase-dependent apoptosis by simultaneous addition of the substance and pan-caspase inhibitor z-VAD-fmk to tube-forming HUVECs. Simultaneous addition of nymphaeol-A and z-VAD-fmk (10 *μ*M) completely blocked nymphaeol-A-induced apoptosis of tube-forming HUVECs (Figure 8). This result strongly suggested that nymphaeol-A-induced cell death in tube-forming HUVECs occurs via caspase-dependent apoptosis.

### 3.5. Inactivation of Survival Signals by Nymphaeol-A

**Figure 9:**
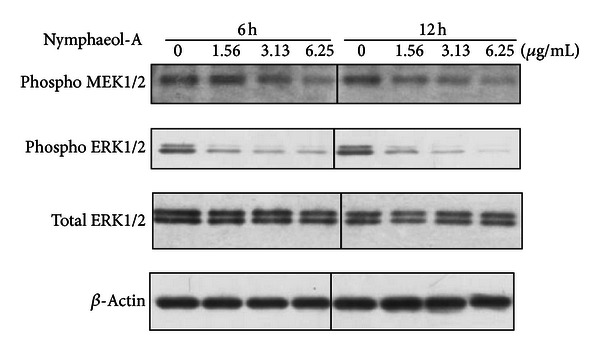
Nymphaeol-A inactivates MEK1/2 and ERK1/2 phosphorylation. Tube-forming HUVECs were treated with 0.4% DMSO (0 *μ*g/mL) or nymphaeol-A at the indicated concentrations for 6 h and 12 h, respectively. Changes in the phosphorylation state of ERK1/2 at Thr202/Tyr204 and MEK1/2 at Ser221 were analyzed by western blotting. Each experiment was repeated at least three times and representative data are shown.

We further analyzed how survival signals were affected by nymphaeol-A using western blotting analysis. Mitogen-activated protein kinase/ERK kinase 1/2 (MEK1/2) is one of the major survival signals in mammalian cells and is known to directly phosphorylate extracellular signal-regulated kinase 1/2 (ERK1/2). Treatment of tube-forming HUVECs with nymphaeol-A (1.56 *μ*g/mL) markedly decreased the phosphorylation of MEK1/2. Subsequently, ERK1/2, which acts downstream of MEK1/2, was also inactivated by nymphaeol-A without any changes in total ERK expression (Figure 9). These observations showed that nymphaeol-A is involved in inhibiting MEK1/2 activation and subsequently inactivates ERK1/2, a cell survival signal in tube-forming HUVECs.

**Figure 10:**
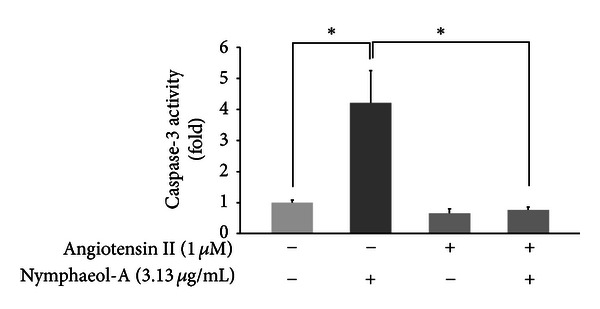
Angiotensin II reverses inactivation of ERK1/2 by nymphaeol-A. HUVECs were pretreated with 1 *μ*M angiotensin II, an ERK1/2 activator, for 3 h. Pretreated cells were incubated with nymphaeol-A at the indicated concentrations for 3 h. Caspase-3/7 activity was measured using the Caspase-Glo Assay Kit by luminescence assays as described in the text. *P < 0.05, as compared with the control group.

To confirm this effect of nymphaeol-A on the MAPK cascade, we used angiotensin II. Angiotensin II, a peptide hormone that causes vasoconstriction and a subsequent increase in blood pressure, is known to activate MAPK and to phosphorylate ERK1/2 in endothelial cells [22]. Treatment of HUVECs with 3.13 *μ*g/mL of nymphaeol-A for 3 h induced caspase-3 activation. Angiotensin II pretreatment, which caused activation of ERK1/2, completely blocked this activation of caspase-3 (Figure 10). This result indicates that angiotensin II inhibits nymphaeol-A-induced apoptosis.

## 4. Discussion

In this study, through our screening program to search for bioactive components with antiangiogenic effects from propolis, we found that the ethanol extracts of Okinawan propolis (EEOP) showed strong antiangiogenic activity *in vitro* and *in vivo*. To find the active components with such antiangiogenic effects, five prenylflavonoids analogs were isolated from EEOP, and nymphaeol-A was investigated for their modes of action of antiangiogenic effect.

We first confirmed that EEOP showed antiangiogenic effects in tube formation assay *in vitro *using tube-forming HUVECs and chorioallantoic membrane (CAM) assay *in vivo* using fertilized chicken eggs. With isolated main components from EEOP, nymphaeols-A and -B had a strong anti-tube-forming activity *in vitro*. Although isonymphaeol-B, 3′-geranyl-naringenin, eriodictyol, and naringenin were shown to inhibit angiogenesis, the effective concentration was higher than that of nymphaeols. In general, it was indicated that the existence of the geranyl moiety was important to express bioactivity [23, 24]. Two of the main components of EEOP, nymphaeols-A and -B, both of which have a prenyl group, showed the strongest anti-tube-forming effect *in vitro* among identified compounds from EEOP. There are some reports which show that remarkable difference of bioactivity arises depending upon the substitution sites of prenyl groups. Particulaly, the existence of prenyl group at C-6 of A ring and catechol structure at B ring of flavonoids was reported to be important for their biological activities such as antioxidative, antifungal, and antimelanogenesis activites [25–28]. Our results corresponded partly with these studies. Furthermore, nymphaeol-A was also found to inhibit angiogenesis *in vivo*. Nymphaeol-B also had antiangiogenic activity *in vivo*, but it was weaker than nymphaeol-A. 

Based on these results, it was concluded that nymphaeol-A had antiangiogenic activities *in vitro* and *in vivo* and could be the primary active component of Okinawan propolis. In addition, these results indicate that propolis from Okinawa might have a tumor-suppressing effect through inhibition of angiogenesis.

We further examined the mechanism of angiogenesis inhibition through nymphaeol-A at the molecular level. Caspase-9, an intrinsic initiator caspase, was activated by nymphaeol-A. Subsequently, caspase-3, an effector caspase, was activated. Treatment with pan-caspase inhibitor z-VAD-fmk was shown to reverse nymphaeol-A-induced caspase-3 activation (Figure 8). These results indicated that nymphaeol-A induces apoptosis in tube-forming HUVECs through the activation of caspase signaling (Figure 7).

MAPK signaling plays important roles in the regulation of angiogenesis, such as stimulation of endothelial cell proliferation, migration, and tube formation [29]. Among MAPK family members, ERKs have been reported to have essential roles in endothelial cell survival and cell migration [30, 31]. Inactivation of these survival signals is known to trigger apoptosis through activation of caspase pathways in endothelial cells [32]. As shown in Figure 9, changes in the level of phosphorylation of MEK1/2 and ERK1/2 were induced in a time- and concentration-dependent manner by nymphaeol-A in HUVECs. Furthermore, Kunimasa et al. revealed that inhibition of MEK1/2 by U0126, MEK1/2 inhibitor, induced apoptosis in HUVECs [32]. These results suggested that caspase activity is regulated by MEK1/2 and ERK1/2 activation in HUVECs. Therefore, our results imply a specific involvement of ERK1/2 and MEK1/2 inactivation in nymphaeol-A-induced apoptosis and its inhibition of angiogenesis. There are some reports that angiotensin II activates the family of mitogen-activated protein kinases (MAPK), including ERK, JNK, and p38 MAPK [33, 34]. Guo et al. observed an effect of angiotensin II on MAPKs in HUVECs and showed that angiotensin II induced phosphorylation of ERK [22]. Pretreatment of HUVECs with angiotensin II completely inhibited nymphaeol-A-induced caspase-3 activation (Figure 10). This result showed that nymphaeol-A acts through the ERK cascade (MEK1/2 and ERK1/2) to subsequently induce apoptosis.

The cell viability of HUVECs in tube formation medium with 5 *μ*g/mL of nymphaeol-A was <10%; however, in growth medium, it was around 60% (data not shown). According to this result, nymphaeol-A may affect tube-forming HUVECs, rather than proliferation-phase HUVECs, by activating the survival signaling. We need to further investigate how nymphaeol-A affects other signaling pathways and induces apoptosis in tube-forming HUVECs at the molecular level. We showed that EEOP and nymphaeol-A inhibit angiogenesis *in vitro* and* in vivo*. Based on these results, nymphaeol-A may represent a new dietary-derived antiangiogenic compound. In addition, these results strongly support the possibility that we can prevent cancer or angiogenesis-related diseases by daily intake of Okinawan propolis which contains nymphaeol-A.
